# Stella-Cre Mice are Highly Efficient Cre Deleters

**DOI:** 10.1002/dvg.20741

**Published:** 2011-02-24

**Authors:** Hui Liu, Wei Wang, Su-Kit Chew, Song-Choon Lee, Juan Li, George S Vassiliou, Tony Green, P Andrew Futreal, Allan Bradley, Shujun Zhang, Pentao Liu

**Affiliations:** 1 College of Animal Science and Technology, Huazhong Agriculture UniversityWuhan, China; 2 Wellcome Trust Sanger InstituteHinxton, Cambridge, CB10 1HH, United Kingdom; 3 Cambridge Institute for Medical Research, University of CambridgeCambridge, CB2 0XY, United Kingdom; 4 Wellcome Trust Sanger InstituteHinxton, Cambridge CB10 1HH, United Kingdom

**Keywords:** *Stella*, *Cre-lox*P recombination, knock-in

## Abstract

Cre-*lox*P recombination is widely used for genetic manipulation of the mouse genome. Here, we report generation and characterization of a new Cre line, Stella-Cre, where Cre expression cassette was targeted to the 3′ UTR of the *Stella* locus. *Stella* is specifically expressed in preimplantation embryos and in the germline. Cre-*lox*P recombination efficiency in Stella-Cre mice was investigated at several genomic loci including *Rosa26*, *Jak2*, and *Npm1*. At all the loci examined, we observed 100% Cre-*lox*P recombination efficiency in the embryos and in the germline. Thus, Stella-Cre mice serve as a very efficient deleter line. genesis 49:689–695, 2011. © 2011 Wiley-Liss, Inc.

## INTRODUCTION

Cre-*lox*P recombination (Sternberg and Hamilton,^1981^) is widely used to modify the mouse genome due to its simplicity and effectiveness. These applications include removing loxP-flanked genomic regions for conditional knockout alleles (Gu *et al*.,^1994^), catalyzing long-range recombination for chromosome engineering and mitotic recombination, and recombination-mediated-cassette-exchange (RMCE) (Herault *et al*.,^1998^; Liu *et al*.,^2002^; Ramirez-Solis *et al*.,^1995^; Smith *et al*.,^1995^; Wallace *et al*.,^2007^; Zong *et al*.,^2005^). To achieve germline Cre-loxP recombination, Cre recombinase can be delivered through several routes. For example, Cre-expression plasmids can directly be injected into fertilized eggs (Araki *et al*.,^1995^; Lauth *et al*.,^2000^; Sunaga *et al*.,^1997^). To avoid plasmid integration, in vitro transcribed Cre mRNA can also be injected into mouse oocytes (de Wit *et al*.,^1998^). However, technical difficulties of microinjection have limited the broad use of Cre/*lox*P system in preimplantation embryos. Recently, it is reported that purified cell-permeable Cre recombinase protein can directly be added to embryo culture media to induce recombination (Kim *et al*.,^2009^).

The development of recombineering technologies allows rapid construction of hundreds of conditional knockout alleles (Chan *et al*.,^2007^; Zhang *et al*.,^1998^). Large genome-wide conditional knockout projects such as EUCOMM and KOMP aim to make conditional knockout alleles for thousands of mouse genes. To study phenotypes of the null alleles of these genes, it is necessary to convert the conditional knockout alleles to null alleles by either expressing Cre in ES cells or using a highly efficient deleter line. It is desirable that the deleter line only expresses Cre transiently in the early embryos to avoid Cre toxicity from prolonged exposure of high levels of Cre recombinase (Schmidt *et al*.,^2000^). On the other hand, Cre expression in the deleter line should be sufficient to induce efficient Cre-loxP recombination. Several germline Cre deleter mouse lines have been produced and used by the mouse genetics community (Hayashi *et al*.,^2003^; Scheel *et al*.,^2003^; Su *et al*.,^2002^; Tang *et al*.,^2002^). These lines however often have mosaic (Scheel *et al*.,^2003^; Su *et al*.,^2002^; Tang *et al*.,^2002^) or sex-specific expression of Cre (Sox2Cre) (Hayashi *et al*.,^2003^), or have Cre expression in adult cells in the case of R26Cre (Soriano,^1999^).

Here, we describe generation and characterization of a new deleter line, Stella-Cre, in the 129Sv/EvBrd (129S5) background. *Stella* (also known as *PGC7*, *Dppa3*) is a maternal effect gene that is specifically expressed in primordial germ cells, oocytes, preimplantation embryos, and ES cells. In post-implantation embryos, Stella is specifically expressed in primordial germ cells (PGCs), and its expression is maintained until E14.5 in the female and E15.5 in the male (Payer *et al*.,^2003^; Saitou *et al*.,^2002^; Sato *et al*.,^2002^). Stella has important functions in early embryo development. Stella-deficient females displayed severely reduced fertility due to a lack of maternally inherited Stella protein in their oocytes. Consequently, embryos from these females rarely reached the blastocyst stage (Payer *et al*.,^2003^).

**FIG. 1:**
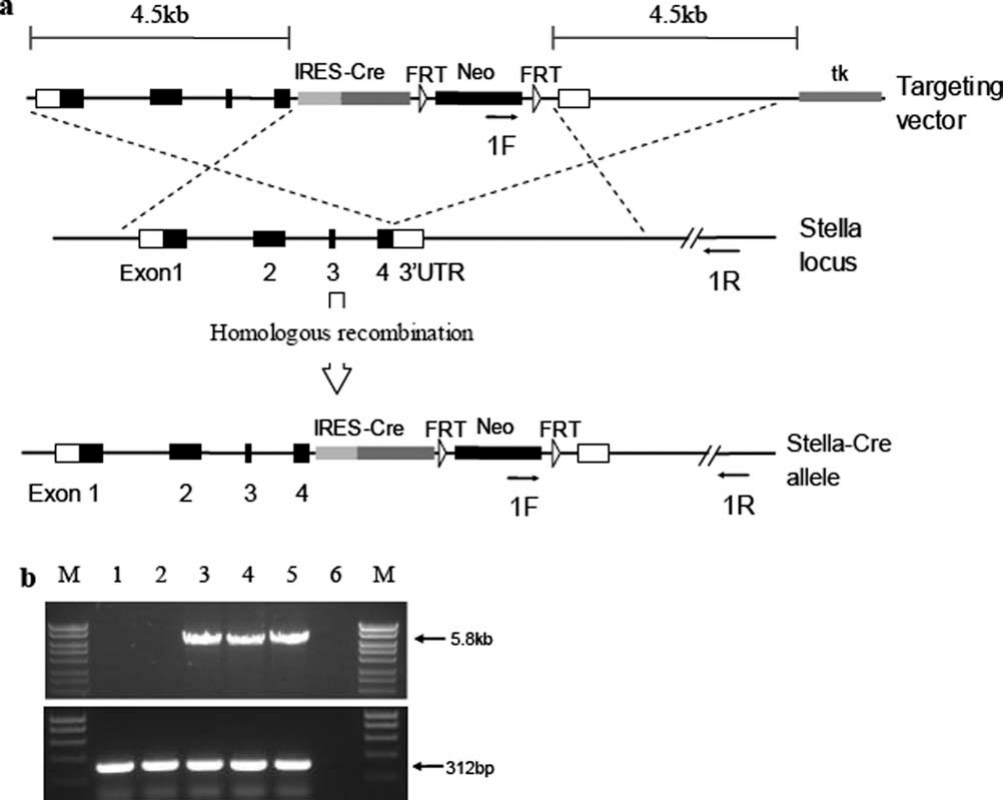
Generation of the *Stella-Cre* allele. (**a**) The *IRES-Cre-FRT-Neo-FRT* cassette was targeted into 3′ UTR just following the stop codon of the *Stella* gene. The targeted clones were genotyped by PCR using primers 1F and 1R. Primer 1R is outside the homology arm. Open boxes at the *Stella* locus: 5′ and 3′ UTRs; Filled boxes: coding regions of *Stella* exons. (**b**) Genotyping of the targeted ES cell colonies. The 5.8-kb junction fragment in the correctly targeted clones was amplified with PCR primers 1F and 1R. Lanes 1–2: two non-targeted ES clones; Lanes 3–5: three targeted clones; Lane 6: negative control (no DNA template). As the PCR control, the 312-bp fragment was amplified from the wild type *Rosa26* locus in all the ES cell clones. M: DNA Marker.

We recombineered the targeting vector from a 129S5 BAC (Adams *et al*.,^2005^) where the *IRES-Cre-FRT-Neo-FRT* cassette was targeted to the 3′ UTR of the *Stella* locus following the stop codon (Fig. 1a). The targeting vector was linearized with NotI and electroporated into AB2.2 ES cells. Transfectants were subsequently selected in G418 for positive and Ganciclovir for negative selections, respectively. Targeted clones were identified by the presence of the 5.8 kb junction fragment using primers from the *Neo* cassette and the genomic DNA outside the homology arms (primers 1F and 1R) (Fig. 1a,b). The correctly targeted ES cell clones were found to express Cre recombinase by RT-PCR analysis and by a reporter assay in which GFP was expressed upon Cre-*lox*P recombination (data not shown). Chimeric mice were produced from two correctly targeted clones. The germline competent chimeras from both ES cell clones were bred to wild type 129S5 females to obtain the Stella-Cre heterozygotes.

**FIG. 2:**
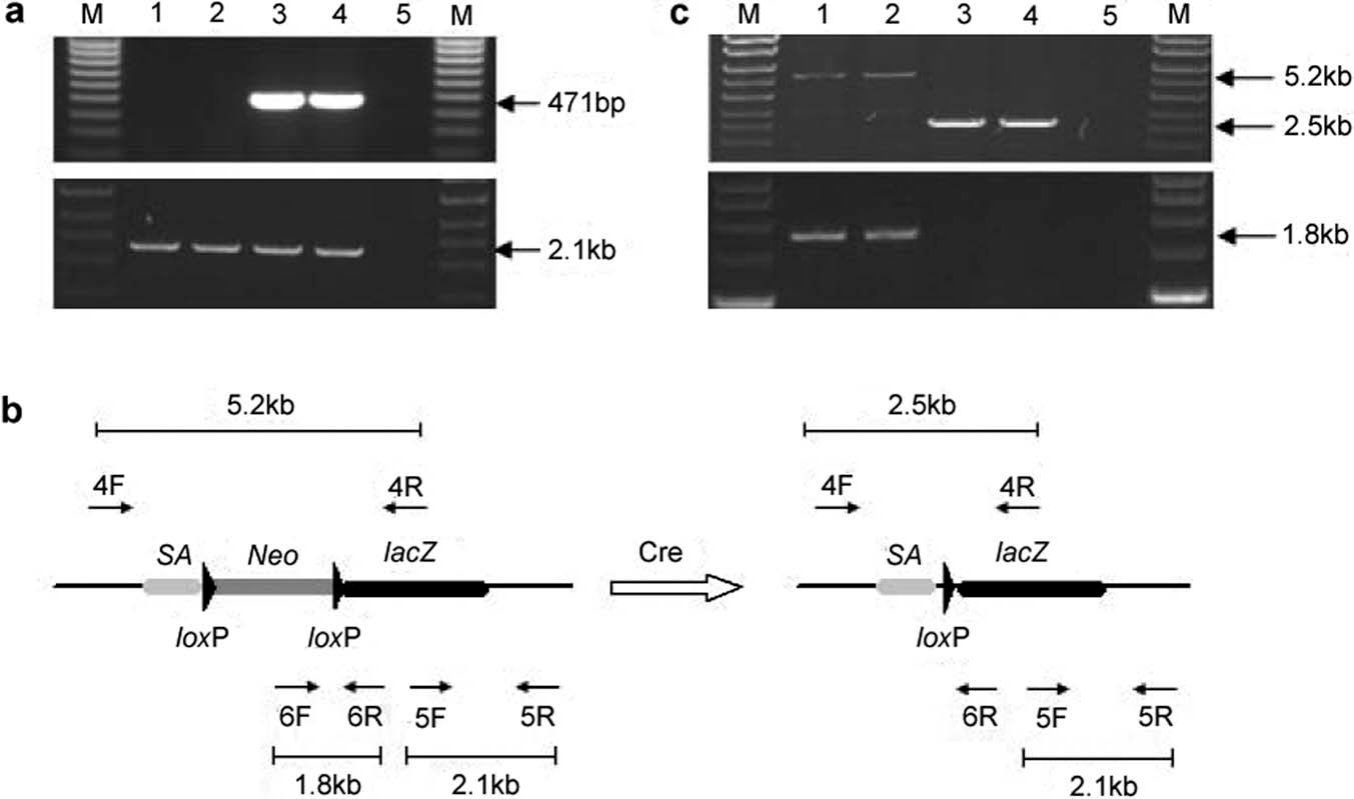
Detection of Cre-*lox*P recombination at the *Rosa26* locus in embryos. Four E12.5 embryos (Lanes 1–4) were harvested from a R26R homozygous female plugged by a Stella-Cre heterozygous male mouse. (**a**) All four embryos inherited the R26R allele which was detected using primers 5F and 5R as the 2.1-kb fragment. PCR analysis also showed that only embryos 3 and 4 had the Stella-Cre allele (471bp Cre fragment). (**b**) Detection of floxed *PGKNeo* cassette excision at the R26R locus. Primers 4F and 4R amplified the 5.2-kb fragment from the non-deletion R26R allele. The size of this fragment was reduced to 2.5 kb after the floxed *PGKNeo* cassette was excised. The non-deletion R26R allele could also be detected using the second pair of primers: 6F and 6R, which amplified a 1.8kb fragment. (**c**) Embryos 1 and 2 did not have the Stella-Cre and thus only had the non-deletion R26R allele as demonstrated by the 5.2-kb PCR fragment with primers 4F and 4R and the 1.8-kb PCR fragment using primers 6F and 6R. Excision of the floxed *PGKNeo* cassette was only detected in Embryos 3 and 4 using primers 4F and 4R as the 2.5-kb fragment. Importantly, we were unable to amplify the 5.2- or 1.8-kb fragments in these two embryos demonstrating that the non-deletion allele was undetectable and that the floxed *PGKneo* cassette was likely excised in most if not all cells. Lane 5: PCR negative control (no DNA template). M: DNA marker.

To test Cre activity *in vivo*, we crossed the Stella-Cre mice to the R26R reporter mouse line (Soriano,^1999^). In the R26R mouse, a floxed stuffer cassette (*PGKNeo*) was inserted in front of the *lacZ* coding sequence at the *Rosa26* locus (Fig. 2b). Once the floxed *PGKNeo* cassette is excised by Cre, *lacZ* expression is activated. We collected embryonic day (E) 12.5 embryos from crosses between Stella-Cre heterozygous males and the homozygous R26R females. Genomic DNA was extracted for genotyping the embryos (Fig. 2a). As anticipated, all embryos had the R26R allele confirmed by the 2.1-kb fragment amplified from the R26R allele (primers 5F and 5R shown in Fig. 2b). Two embryos (Lanes 3 and 4) also inherited the Stella-Cre allele as shown by the 471 bp Cre specific amplification fragment (Fig. 2a). Cre-*lox*P recombination was expected to excise the floxed *PGKNeo* cassette. The non-deletion R26R allele was detected using primers 4F and 4R as a 5.2 kb fragment, or a 1.8-kb fragment amplified using primers 6F and 6R (Fig. 2b). After *PGKNeo* excision, primers 4F and 4R amplified a 2.5-kb fragment from the deletion R26R allele (Fig. 2b). As shown in Figure 2c, embryos 1 and 2 did not have any excision since they did not have the 2.5-kb band, while efficient deletion was found in embryos 3 and 4. Importantly, in the Stella-Cre/R26R embryos (Lanes 3 and 4, Fig. 2c), we did not detect any non-deletion allele band (5.2 kb) even after several attempts, indicating very efficient excision of the floxed *PGKNeo* cassette at the *Rosa26* locus at the time of detection (E12.5). This result was confirmed by using primers 6F and 6R which amplified the smaller non-deletion fragment (1.8 kb) only from embryos 1 and 2 but not from 3 and 4 (Fig. 2c). Besides embryos, complete Cre-*lox*P deletion was also found in the extraembryonic tissues (yolk sac) (data not shown), consistent with zygotic Stella expression starting in the 2-cell stage embryo (Payer *et al*.,^2003^).

**FIG. 3:**
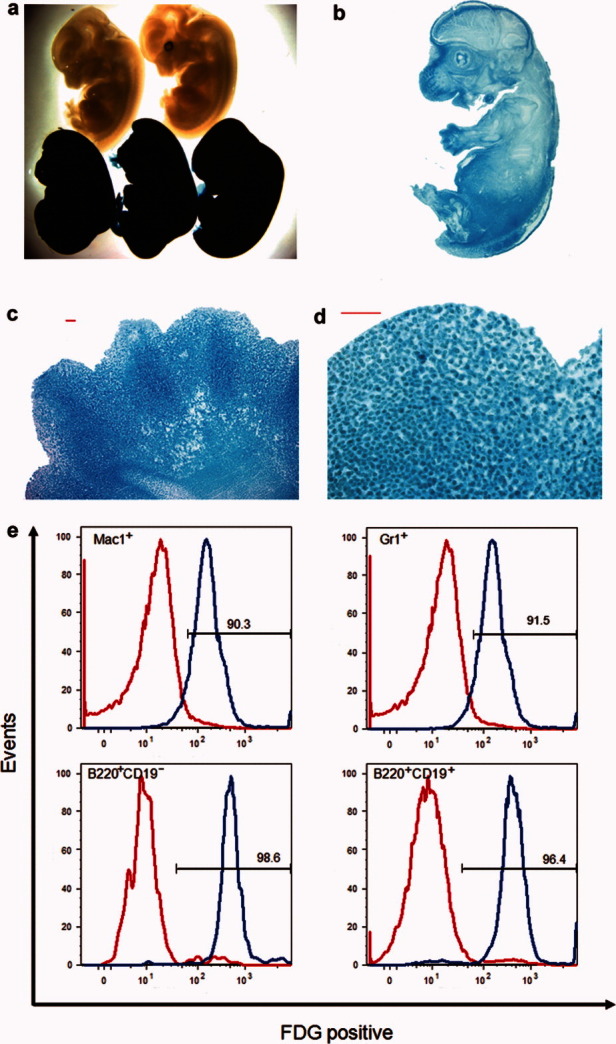
Efficient Cre-*lox*P recombination at the *Rosa26* locus (R26R allele) detected by lacZ activities. (**a**) Whole-mount X-gal staining of Stella-Cre/R26R compound heterozygous (Blue ones) or R26R only (non-blue ones) E12.5 embryos. (**b**) Sagittal section of an E15.5 Stella-Cre/R26R compound heterozygous embryo. (**c**,**d**) Higher magnification sagittal sections showing lacZ expression in all cells in the forelimb. Scale bars, 50 μm. (**e**) Flow cytometric analysis of bone marrow cells from Stella-Cre/R26R mice to detect Cre-*lox*P recombination by staining *lacZ*-expressing cells using FDG. In the FACS profiles, the red line represents the wild type control cell staining while the blue line depicts the lacZ-FDG signal in Stella-Cre/R26R cells. Almost all cells of Stella-Cre/R26R were stained positively for FDG and thus expressed lacZ.

To further demonstrate the efficient deletion in the R26R reporter mice, we stained the embryos heterozygous for both Stella-Cre and R26R alleles with X-Gal. These embryos displayed ubiquitous blue staining (Fig. 3a). In contrast, no lacZ activity was found in the R26R embryos without the Cre (Fig. 3a). Sectioning of the compound heterozygous embryos confirmed that *lacZ* was expressed in all cells of the embryo (Fig. 3b–d).

We next examined Cre-*lox*P recombination at a single cell level in Stella-Cre/R26R double transgenic mice taking advantage of the fluorescent substrate of β-galatosidase fluorescein di-β-d-galactopyranoside (FDG). Bone marrow cells from the Stella-Cre/R26R mice were harvested and stained briefly with FDG before subjected to flow cytometric analysis. Robust FDG staining was found in all the examined hematopoietic lineages: 92% in Gr1^+^ cells, 90% in Mac1^+^, 99% in B220^+^CD19^−^ early B cells, 96% in B220^+^CD19^+^ B cells (Fig. 3e). The lower than 100% FDG staining in bone marrow cells was expected due to the relatively low sensitivity of FDG in some blood cells. This possibility was confirmed in PCR analysis where we did not detect any undeleted fragment of the R26R allele using genomic DNA of the sorted cells (data not shown).

*Stella* is expressed in early germline development. To directly determine Cre-*lox*P recombination in the germline, we bred the double transgenic males that had both the Stella-Cre allele and a floxed *eGFP* cassette at the *Rosa26* locus to wild type females. Twenty pups were found to inherit only the modified *Rosa26* allele. The *eGFP* cassette was excised in all twenty pups thus demonstrating 100% germline Cre-*lox*P recombination in the parental male mice (data not shown). Both male and female Stella-Cre mice were subsequently crossed to conditional knockout or knock-in mice of other loci including *Jak2* and *Npm1*. In all cases, we did not detect the non-recombination fragments in PCR analysis in E12.5-14.5 embryos or in adult mice that had both the Cre and the floxed loci (data not shown), further confirming the efficient Cre-*lox*P recombination by the Stella-Cre. No noticeable difference in recombination efficiency was observed between using male and female Stella-Cre mice. Additionally, excision of the FRT-flanked *Neo* cassette from the Stella-Cre allele did not appear to affect the Cre-*lox*P recombination efficiency (data not shown).

In summary, we have generated and characterized the Stella-Cre mouse line. Consistent with Stella's expression primarily in early embryos and in the germline, Stella-Cre led to very efficient Cre-*lox*P recombination in embryos and in the germline examined at several loci. Stella-Cre mice thus serve as a highly efficient Cre deleter line. Compared to other germline deleters used in the community, Stella-Cre mice offer the following advantages: *Stella* expression is primarily restricted to the germline and preimplantation embryos; Stella-Cre is a knock-in allele thus it is likely that Cre activity recapitulates the endogenous Stella expression so that Cre-loxP recombination is primarily restricted to preimplantation embryos and the germline. Moreover, although Stella was considered to be a maternal effect gene, zygotic Stella expression starts as early as in the two-cell stage embryo (Payer *et al*.,^2003^). Additionally, Stella is expressed during both male and female germline development. Therefore, both male and female Stella-Cre mice can be used as very efficient deleters.

## MATERIALS AND METHODS

### Construction of Stella-Cre Targeting Vector

To make the retrieving vector, the 5′ and 3′ retrieving homology arms of *Stella* gene region were amplified using PCR from mouse BAC (bMQ-301M14) with the following primers:

5′ HA forward: *Stella-out-5F-NotI*: 5′TTgcggccgcGTTGTGCGGATGGTGTTGTAAGCTTT-3′,

5′ HA reverse: *Stella-out-5R-BamHI*: 5′-CGggatccCCACCCCCTTCCCATCAGTTAAGTTAA-3′),

3′ HA Forward: *Stella-out-3F-BamHI-SpeI*: 5′-CGggatccactagtGCTGGTCTGTTCTGCTAAGTAGTCAGG-3′,

3′ HA Reverse: *Stella-out-3R-XbaI*:

5′-GtctagaCTCTGATTCATTGGATCAGCAGCC-3′

The 5′ and 3′ retrieving homology arms were cut with NotI/BamHI and BamHI/XbaI, respectively before cloned into the pL253 vector (Liu *et al*.,^2003^) to construct the retrieving vector. The retrieving vector was linearized with BamHI/SpeI to retrieve the 9.0 kb genomic fragment from BAC (bMQ-301M14).

For the mini-targeting vector, the 5′ and 3′ homology arms (HA) were amplified using the following primers:

5′ HA Forward: *Stella-in-5F-SalI*: 5′-CGgtcgacCTCAGCCCCCAGGAAGTCTGGT-3′, 5′ HA Reverse: *Stella-in-5R-EcoRI*: 5′-CGgaattcCGACAGCCAGGGCAGCGTAC-3′, 3′ HA Forward: *Stella-in-3F-BamHI*: 5′-CGggatccCGACGATGCCGCACAGCAG-3′,

3′HAReverse:*Stella-in-3R-NotI*:

5′ TTgcggccgcCTGTTACAGTAGCCCCTAGCTGTTTGTG −3′.

The 5′ and 3′ homology arms were cut with EcoRI/SalI and BamHI/NotI, respectively. The *IRES-Cre-FRT-PGK-Neo-FRT* fragment was cut out from pL459 vector using EcoRI/BglII. The homology arms and the Cre cassettes were cloned into the pBlueScriptII SK(+) cut with with SalI/NotI to obtain the mini-targeting vector. The two homology arms and the Cre cassette was finally cut out as one fragment using XhoI/NotI which was electroporated into the retrieved vector for recombineering to obtain the final targeting vectors.

### Generation of Stella-Cre Mice

The NotI-linearized Stella-IRES-Cre-FRT-Neo-FRT targeting vector was electroporated into AB2.2 ES cells, which were cultured using standard protocols (Ramirez-Solis *et al*.,^1993^). G418 and Ganciclovir double resistant colonies were picked and expanded for genotyping using the following primers:

1F: 5′ GAGGAAATTGCATCGCATTGTCTGAGTAGG 3′

1R: 5′ GCGACTTACAGAATGTGAAGTTAGGCAGC 3′

Two correctly targeted ES cell clones were injected into C57/BL6 blastocysts to obtain chimeras. Mice tail tip DNA was extracted and genotyped by PCR analysis using Cre specific primers (2F: 5′ CCGGTCGATGCAACGAGTGATGAGGTT 3′,

2R: 5′ CAGGGTGTTATAAGCAATCCCCAGAAATGCC3′) and

*Rosa26* primers (3F: 5′AAAGTCGCTCTGAGTTGTTAT3′, 3R: 5′ GCGAAGAGTTTGTCCTCAACC 3′).

All animal experiments were performed in accordance with the UK's 1986 Animals Scientific Procedure Act and local institute ethics committee regulations.

### Genotyping Embryos and Whole-Mount X-Gal Staining

E12.5 embryos were obtained from crosses between Stella-Cre/+ males and R26R/R26R females. Embryos were genotyped by PCR using both Cre primers (2F: 5′ CCGGTCGATGCAACGAGTGATGAGGTT 3′, 2R: 5′ CAGGGTGTTATAAGCAAT CCCCAGAAATGCC 3′) and *lacZ* (5F: 5′ AGAGACGCGCCCGCTGATCC 3′, 5R: 5′ GGAGCGGGAGAAATGGATATG 3′). Cre-mediated excision was determined using two pairs of primers: one pair is (4F: 5′ AAAGTCGCTCTGAGTTGTTAT 3′, 4R: 5′CAGGCGCTGATGTGCCCGGC 3′) and the other is (6F: 5′ GCCGCTTTTCTGGATTCATCGA 3′, 6R: 5′ GGCCTCAGGAAGATTGCACTCCA 3′). Dissected embryos were fixed in 4% paraformaldehyde at 4°C for 15 min. After washing three times with ice cold PBS, embryos were incubated in lacZ staining buffer (0.1% X-Gal (1 mg ml^−1^) in DMF, 2 mM MgCl_2_·6H_2_O, 0.01% deoxycholic acid, 0.02% IGEPAL CA-630, 5 mM potassium ferrocyanide (K_4_Fe(CN)_6_·3H_2_O), and 5 mM potassium ferricyanide [K_4_Fe(CN)_6_ in PBS (pH 8.0)] for up to 48 h in the dark at 4°C.

### Flow Cytometric Analysis of Hematopoietic Cells Using FDG

Fluorescein di-β-d-galactopyranoside (FDG, Sigma) was used for flow cytometric analysis of the deletion efficiency in bone marrow cells. Long bones (tibias and femurs) were removed from 8- to 12-week old mice. Cells were stained on ice in the dark for 20 min using the following monoclonal antibodies: Gr-1, Mac1, B220, CD19 (all from BD Biosciences). About 1.0 ml PBS (with 1% FCS) was then add and cells were centrifuged at 375*g* for 5 min. Supernatant was then decanted and cells were re-suspended in 500 μl of PBS (with 1% FCS). Cell samples and FDG stock (2.0 mM in DMSO) were pre-warmed at 37°C for 5 min. Next, an equal volume of FDG stock was added and the cell mixture was incubated at 37°C for 1 min before adding 2.0 ml of HBSS for one hour incubation on ice in dark. Flow cytometric analysis was carried out using CyAN ADP (DakoCytomation) or MoFlo (DakoCytomation) and gates were set to exclude >99.9% of cells labeled with isoform-matched control antibodies conjugated with the corresponding fluorochromes.

## References

[b1] Adams DJ, Quail MA, Cox T, van der Weyden L, Gorick BD, Su Q, Chan WI, Davies R, Bonfield JK, Law F, Humphray S, Plumb B, Liu P, Rogers J, Bradley A (2005). A genome-wide, end-sequenced 129Sv BAC library resource for targeting vector construction. Genomics.

[b2] Araki K, Araki M, Miyazaki J, Vassalli P (1995). Site-specific recombination of a transgene in fertilized-eggs by transient expression of cre recombinase. Proc Natl Acad Sci USA.

[b3] Chan W, Costantino N, Li R, Lee SC, Su Q, Melvin D, Court DL, Liu P (2007). A recombineering based approach for high-throughput conditional knockout targeting vector construction. Nucleic Acids Res.

[b4] de Wit T, Drabek D, Grosveld F (1998). Microinjection of Cre recombinase RNA induces site-specific recombination of a transgene in mouse oocytes. Nucleic Acids Res.

[b5] Gu H, Marth JD, Orban PC, Mossmann H, Rajewsky K (1994). Deletion of a DNA polymerase beta gene segment in T cells using cell type-specific gene targeting. Science.

[b6] Hayashi S, Tenzen T, McMahon AP (2003). Maternal inheritance of Cre activity in a Sox2Cre deleter strain. Genesis.

[b7] Herault Y, Rassoulzadegan M, Cuzin F, Duboule D (1998). Engineering chromosomes in mice through targeted meiotic recombination (TAMERE). Nat Genet.

[b8] Kim K, Kim H, Lee D (2009). Site-specific modification of genome with cell-permeable Cre fusion protein in preimplantation mouse embryo. Biochem Biophys Res Commun.

[b9] Lauth M, Moerl K, Barski JJ, Meyer M (2000). Characterization of Cre-mediated cassette exchange after plasmid microinjection in fertilized mouse oocytes. Genesis.

[b10] Liu PT, Jenkins NA, Copeland NG (2002). Efficient Cre-loxP-induced mitotic recombination in mouse embryonic stem cells. Nat Genet.

[b11] Liu PT, Jenkins NA, Copeland NG (2003). A highly efficient recombineering-based method for generating conditional knockout mutations. Genome Res.

[b12] Payer B, Saitou M, Barton SC, Thresher R, Dixon JPC, Zahn D, Colledge WH, Carlton MBL, Nakano T, Surani MA (2003). Stella is a maternal effect gene required for normal early development in mice. Curr Biol.

[b13] Ramirez-Solis R, Davis AC, Bradley A (1993). Gene targeting in embryonic stem cells. Methods Enzymol.

[b14] Ramirez-Solis R, Liu P, Bradley A (1995). Chromosome engineering in mice. Nature.

[b15] Saitou M, Barton SC, Surani MA (2002). A molecular programme for the specification of germ cell fate in mice. Nature.

[b16] Sato M, Kimura TU, Kurokawa K, Fujita Y, Abe K, Masuhara M, Yasunaga T, Ryo A, Yamamoto M, Nakano T (2002). Identification of PGC7, a new gene expressed specifically in preimplantation embryos and germ cells. Mech Dev.

[b17] Scheel JR, Garrett LJ, Allen DM, Carter TA, Randolph-Moore L, Gambello MJ, Gage FH, Wynshaw-Boris A, Barlow C (2003). An inbred 129SvEv GFPCre transgenic mouse that deletes loxP-flanked genes in all tissues. Nucleic Acid Res.

[b18] Schmidt EE, Taylor DS, Prigge JR, Barnett S, Capecchi MR (2000). Illegitimate Cre-dependent chromosome rearrangements in transgenic mouse spermatids. Proc Natl Acad Sci USA.

[b19] Smith AJ, De Sousa MA, Kwabi-Addo B, Heppell-Parton A, Impey H, Rabbitts P (1995). A site-directed chromosomal translocation induced in embryonic stem cells by Cre-loxP recombination. Nat Genet.

[b20] Soriano P (1999). Generalized lacZ expression with the ROSA26 Cre reporter strain. Nat Genet.

[b21] Sternberg N, Hamilton D (1981). Bacteriophage-P1 site-specific recombination. 1. Recombination between Loxp sites. J Mol Biol.

[b22] Su H, Mills AA, Wang XZ, Bradley A (2002). A targeted X-linked CMV-Cre line. Genesis.

[b23] Sunaga S, Maki K, Komagata Y, Ikuta K, Miyazaki J (1997). Efficient removal of loxP-Flanked DNA sequences in a gene-targeted locus by transient expression of Cre recombinase in fertilized eggs. Mol Reprod Dev.

[b24] Tang SHE, Silva FJ, Tsark WMK, Mann JR (2002). A Cre/loxP-deleter transgenic line in mouse strain 129S1/SvImJ. Genesis.

[b25] Wallace HA, Marques-Kranc F, Richardson M, Luna-Crespo F, Sharpe JA, Hughes J, Wood WG, Higgs DR, Smith AJ (2007). Manipulating the mouse genome to engineer precise functional syntenic replacements with human sequence. Cell.

[b26] Zhang Y, Buchholz F, Muyrers JP, Stewart AF (1998). A new logic for DNA engineering using recombination in *Escherichia coli*. Nat Genet.

[b27] Zong H, Espinosa JS, Su HH, Muzumdar MD, Luo L (2005). Mosaic analysis with double markers in mice. Cell.

